# Lung ultrasound in children and adolescents with long-term effects of COVID-19: Initial results

**DOI:** 10.3389/fped.2023.1112881

**Published:** 2023-03-24

**Authors:** Stephanie Gräger, Rosalie Pfirschke, Michael Lorenz, Daniel Vilser, Martin Krämer, Hans-Joachim Mentzel, Katja Glutig

**Affiliations:** ^1^ Department of Diagnostic and Interventional Radiology, University Hospital Jena, Jena, Germany; ^2^ Cystic Fibrosis Center for Children and Adults, University Hospital Jena, Jena, Germany; ^3^ Cardiology Section, Department of Child and Adolescent Medicine, University Hospital Jena, Jena, Germany; ^4^ Section Pediatric Radiology, Department of Diagnostic and Interventional Radiology, University Hospital Jena, Jena, Germany

**Keywords:** follow-up, COVID Syndrome, pulmonary function, diagnostic Lung sonography, complication, screening

## Abstract

**Introduction:**

Chronic health effects following acute COVID-19 are increasingly observed as the pandemic continues and are grouped under long COVID. Although the acute course of the COVID disease is often milder, long COVID also affects children and adolescents. As the symptoms present in Long-COVID often seem to be non-specific and not limited to organ systems, clarification of the causes and the creation of a meaningful, efficient and targeted diagnostic algorithm is urgently needed.

**Methods:**

Therefore, in this prospective observational study, we examined 30 children with long COVID using lung ultrasound and compared the results with those of 15 lung-healthy children.

**Results:**

In our study, no significant difference was found between the two groups in the morphological criteria of lung ultrasound of the pleura or pleural lung structures. There was no significant correlation between the lung ultrasound findings and clinical Data.

**Discussion:**

Our findings are congruent with the current, albeit sparse, data. It is possible that the causes of persistent thoracic symptoms in long COVID might be more likely to be present in functional examinations, but not morphologically imageable. Nonspecific symptoms do not appear to be due to changes in the lung parenchyma. In conclusion, lung ultrasound alone and without baseline in acute disease is not suitable as a standard in the follow-up of long COVID patients. Further investigations on the morphological and functional changes in patient with long COVID is needed.

## Introduction

The pandemic coronavirus disease (COVID-19) has been a global health hazard for most of the last 3 years. While until today children and adolescents usually suffer less in cases of acute COVID-19 infection, 2%–3.5% of primarily non-hospitalized children show long-term effects (1) after the coronavirus infection such as fatigue, muscle weakness, dyspnea, and neurological problems more than 4 weeks later, also known as long COVID (2). In adults, Wang et al. described residual findings in lung-CT in up to 94% of the patients after 14 days after initial symptoms (3). While follow-up of adults with long COVID by CT seems feasible and reasonable (4), a very strict indication for the use of x-rays in pediatric patients is indicated because of its potentially adverse effects (5). In recent years, lung ultrasound (LUS) has emerged as an effective, non-invasive, and radiation free diagnostic modality for superficial localized interstitial lung disease (6). Lung ultrasound (LUS) has also been shown to be useful for the diagnosis of COVID-19 in the acute stage of the disease (7). In adults, the usefulness of control examinations with LUS has already been demonstrated (6). To date, there seems to be a lack of experience with LUS in pediatric long COVID patients. It is unclear whether LUS is an effective follow-up method, even in patients without initial pneumonia. In this study, LUS was used to examine children and adolescents with long COVID to clarify whether there were significant LUS findings in this patient group.

## Methods

### Ethics statement

Ethical approval was obtained prior to the start of the study by the local Medical Ethics Committee [Local approval Number 2021-2300-DATA]. Informed consent was obtained from the custodial parents of all study participants, and written informed consent was obtained from all patients or their parents. Subsequent analysis of ultrasound images was performed as part of the BMBF-funded LongCOCid study (BMBF number 01EP2101A). All procedures performed in this study involving human participants were conducted in accordance with the ethical standards of the institutional and national research committees and with the 1964 Helsinki Declaration and its later amendments, or comparable ethical standards.

### Study design and cohort

Between May and October 2021, 30 patients with long COVID were prospectively enrolled in this observational single-center study. Male and female patients between 5 and 18 years of age were included only if COVID-19 infection was detected by on-site laboratory chemistry.

In the healthy control group, children of both sexes between the ages of 5 and 18 years were included.

The long COVID group included 16 girls (53.3%) and 14 boys (46.7%) with a mean age of 12.9 years with a standard deviation (SD) of 3.6 years. The long COVID group consisted of both, post COVID and long COVID patients according to the NICE-Guidelines (8). The clinical and descriptive data of the participants in the long COVID group and control group are presented in Table 1.

**Table 1:** Clinical and descriptive data of the participants in the study and control group.

Parameters	Long COVID group*	Control group*
Age in years	12.3 ± 3.8	11 ± 3.3
Height in cm	157.5 ± 16.5	152.4 ± 15.5
Weight in kg	52.3 ± 17.9	43.9 ± 12.7
BMI in kg/m^2^	20.6 ± 4.6	18.6 ± 3.6
Heartrate in bpm	85 ± 15	
Blood pressure in mmHg	113.2 ± 12.1/67.4 ± 9.1	
FVC in %	106.4 ± 13.5	
FEV in %	105 ± 13.3	
MEF 25 in %	118.6 ± 48.4	
Time since acute COVID infection in months	4.6 ± 3.3	

BMI, body-mass-index; FVC, forced vital capacity; FEV, forced expiratory volume; MEF, maximal expiratory flow.

* Numerical values are mean with standard deviation (SD).

The control group included children and adolescents without a history of any pulmonary disease, who underwent abdominal ultrasound due to nocturia or chronic abdominal pain in our department. There were 8 (53.3%) girls and 7 (46.7%) boys with a mean age of 11 years and an SD of 3.3 years.

### Lung ultrasound

**Figure 1:**
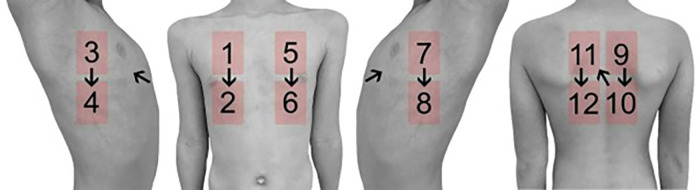
Schematic illustration of the examined localization in lung ultrasound.

Ultrasound examination was performed in accordance with the international consensus (9). Ultrasound was performed using a 10 MHz probe of a Siemens Sequoia Ultrasound system (Erlangen, Germany). Twelve different positions on the thorax were examined, consisting of two ventral, two lateral and two dorsal localizations each on the left and right hemithorax. The different localizations are shown in Figure 1. At each of these localizations, an image was acquired in in- and expiration, both longitudinally (sagittal view) and transversely (axial view) to the intercostal space. Therefore, 48 images were acquired for each patient. Each examination lasted for approximately 12 min.

**Figure 2:**
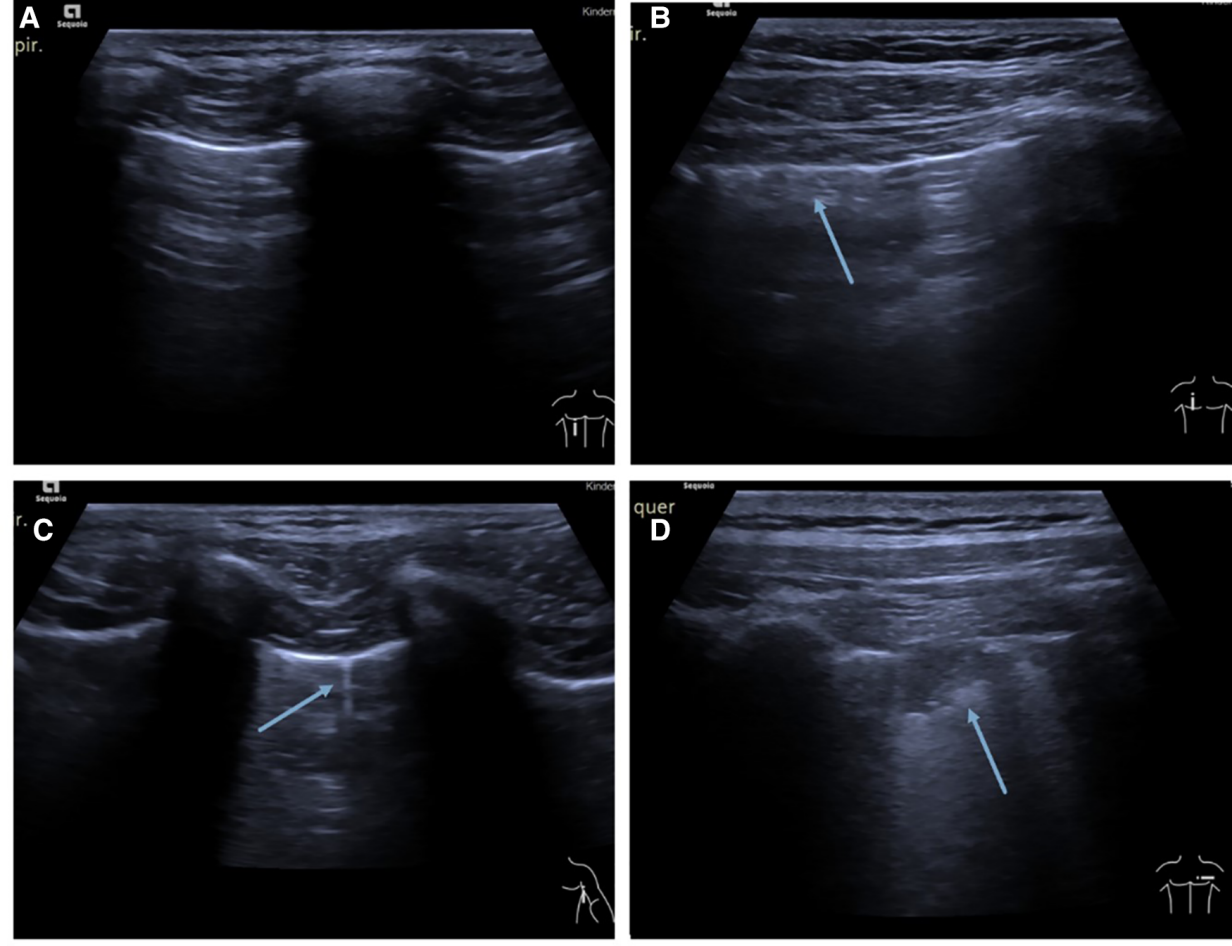
(**A**) Example of a normal lung ultrasound. Picture showing a typical A-line pattern. (**B**) Lung ultrasound picture showing pleural irregularity (arrow). (**C**) Example of a typical B-line (arrow). (**D**) Example picture of a typical consolidation (arrow).

Subsequently, all 2,160 images were anonymized in Microsoft Photos (version 2021.21090.10008.0) and randomized Microsoft Excel (Microsoft Corporation, 2018). All anonymized and randomized images were assessed independently by two readers according to predefined criteria. Reader 1 had approximately 20 years of experience in pediatric radiology and ultrasound, while reader 2 had approximately 1-year of experience in pediatric radiology and ultrasound. For image assessment the following criteria were predefined according to Volpicelli (10): B-lines, consolidations, pleural irregularities, and pleural effusions were detectable on the images (Figure 2). Comet tail artifacts and B-lines were classified into one group. The number of B-lines in the image were counted out. In the case of consolidation, pleural irregularity, or pleural effusions, this was scored as 1 and 0 if they were not present.

### Pulmonary function testing and ECG

Patients with long COVID underwent a standard spirometry program in the Jaeger Masterscreen pneumotachograph spirometer (CareFusion, Yorba Linda, CA, United States) (11) and multimodal transthoracic echocardiogram (TTE) with an iE33 echocardiography system (Royal Philips Electronics, Amsterdam, Netherlands). Echocardiographic measurements were performed according to current guidelines of the German Society of Pediatric Cardiology (12).

### Statistical analysis

Reclassification and statistical analysis (IBM SPSS 28) were performed. Demographic data were assessed with descriptive statistics. For statistical analysis of group differences, a Mann-Whitney *U* test was applied to every lung ultrasound criterion (B-lines, consolidations, pleural effusion, and pleural irregularity) and presented in boxplots by exploratory analysis. In addition, receiver operating characteristic (ROC) curves of each feature were made to determine thearea under the curve (AUC). Correlations between clinical parameters (number of symptoms, pathological lung function or single lung function parameters, heart rate) and single criteria of lung ultrasound were proven by Spearman correlation. Inter-reader reliability was calculated on 2,160 pictures with three diagnostic criteria each using Cohen’s kappa.

## Results

30 patients between the ages of 5–18 years with persistent symptoms after COVID infection were studied over a period of 6 months. In addition, 15 patients without lung-specific symptoms were examined as a comparison population during the same period using the same ultrasound protocol.

With 66.6% fatigue and 23.3% concentration difficulties were the most common complaints in the study group. Only 13.3% of the long COVID patients showed respiratory symptoms. Detailed data on the symptoms of the study group are presented in Table 2.

**Table 2:** Description of symptoms in the long COVID group.

Symptom	Data*
**Respiratory symptoms**	
Dyspnoea	4/30 (13.3%)
Cough	4/30 (13.3%)
**Cardiovascular symptoms**	
Orthostatic dysfunction	2/30 (6.6%)
Dizziness	2/30 (6.6%)
Positional vertigo	1/30 (3.3%)
**Generalised symptoms**	
*Fatigue*	20/30 (66.6%)
PIMS	3/30 (10%)
Fever	1/30 (3.3%)
**Neurological symptoms**	
Headache	3/30 (10%)
**Gastrointestinal symptoms**	
Abdominal pain	3/30 (10%)
Diarrhoea	2/30 (6.6%)
Nausea	1/30 (3.3%)
Gastroenteritis	1/30 (3.3%)
Vomiting	1/30 (3.3%)
**Musculoskeletal symptoms**	
Myalgia	2/30 (6.6%)
Joint pain	2/30 (6.6%)
**Ear, nose, and throat symptoms**	
Loss of taste and/or smell	3/30 (10%)
Parosmia	2/30 (6.6%)
**Psychological/psychiatric symptoms**	
Concentration difficulties	7/30 (23.3%)
Sleep difficulties	2/30 (6.6%)
Anxiety	2/30 (6.6%)

PIMS, paediatric inflammatory multisystem syndrome; MISC, multisystem inflammatory syndrome in children.

* Data are presented as *n*/*N* (%).

Patients in the control group presented mainly because of persistent nocturia (Table 3). Acute Covid infection in the long COVID group was on average 4.6 months ago. Most of the long COVID patients (86.7%) showed no or only mild Symptoms in acute COVID infection. One (3.3%) of the long COVID Patients had been hospitalized during acute COVID infection, 3 Patients developed symptoms of PIMS (6.6%). In ony 10% of the patients with long COVID a pathological pulmonary function was detected and 6.6% of the patients showed pathological echocardiography, none of them both.

**Table 3:** Description of symptoms in the control group.

Symptom	Data>*
**Nephrological symptoms**	
Follow-up assessment^a^	3/15 (20%)
Enuresis nocturna	1/15 (6.6%)
**Generalised symptoms**	
Fatigue	1/15 (6.6%)
**Gastrointestinal symptoms**	
Follow-up assessment^a^	4/15 (26.6%)
Abdominal pain	3/30 (20%)
Diarrhoea	1/30 (6.6%)
Hematemesis	1/30 (6.6%)
**Endocrine symptoms**	
Follow-up assessment^a^	1/30 (6.6%)
**Psychological/psychiatric symptoms**	
Lack of weight gain	1/30 (6.6%)

* Data are presented as *n*/*N* (%).

^a^ Asymptomatic follow-up assessment in the context of the pre-existing conditions.

LUS could be performed in all patients with long COVID as well as all controls sufficiently. All acquired images could be evaluated adequately, as a quality control was already carried out by the examiner during the acquisition and any insufficient images were retaken. For better comprehensibility and clarity and given substantial inter-reader reliability with a Cohen’s kappa of 0.67, only results from one reader are presented below.

**Figure 3:**
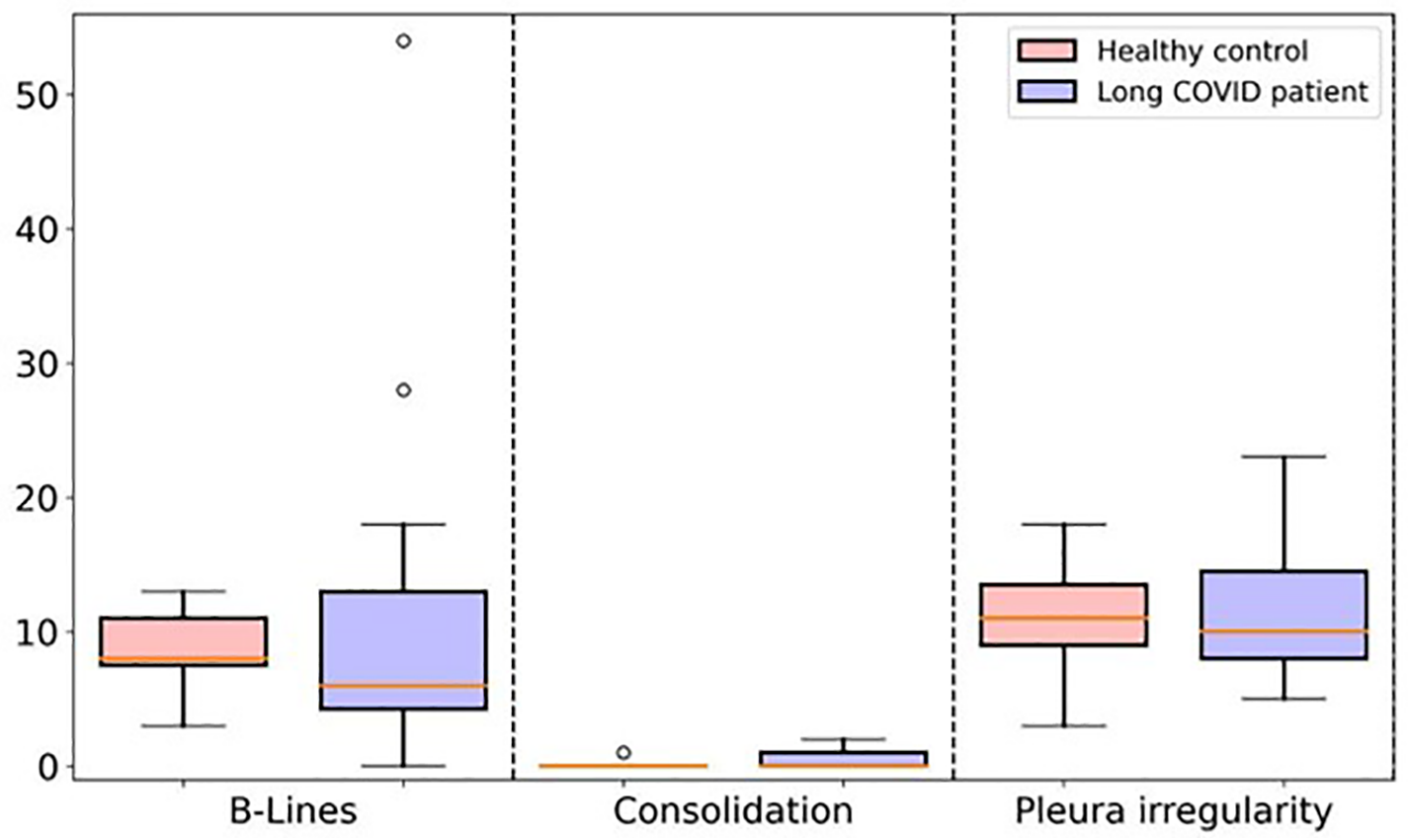
Boxplot comparing the total number of B-lines, consolidations, and pleural irregularity between long COVID study group and healthy control group.

Statistical analysis showed no significant differences between long COVID patients and healthy children in our groups in the total number of B-lines (*p* 0.393), consolidations (*p* 0.077), or pleural irregularities (*p* 0.078) over all localization for each patient (Figure 3). Pleural effusions were not detected in any of the children examined.

There were no significant correlations between clinical features (*Z*-Score in LUFU for small airway obstruction and restriction and general obstruction and restriction, pathological echocardiography, heartrate, or number of symptoms) and LUS features (Table 4).

**Table 4.** Statistical correlation of clinical parameters with LUS features.

		B-lines	Consolidations	Pleural irregularity
**Obstruction**	Correlation coefficient	−0.359	−0.203	−0.080
	p-value	0.092	0.352	0.0716
			
**Restriction**	Correlation coefficient	−0.182	−0.292	−0.123
	p-value	0.405	0.177	0.575
			
**Small airway obstruction**	Correlation coefficient	0.047	0.187	−0.041
	p-value	0.829	0.381	0.851
			
**Small airway restriction**	Correlation coefficient	0.154	−0.108	0.284
	p-value	0.570	0.689	0.287
			
**Pathological ECG**	Correlation coefficient	−0.201	−0.174	0.132
	p-value	0.286	0.358	0.488
			
**Heartrate**	Correlation coefficient	−0.011	0.028	−0.173
	p-value	0.954	0.882	0.360
			
**Number of symptoms**	Correlation coefficient	0.015	0.006	−0.071
	p-value	0.938	0.974	0.711

## Discussion

The results of quantitative analysis found no significant differences in lung ultrasound between children with long COVID and the healthy control group. This is consistent with recently published articles. Denina et al. reported complete normalization of lung ultrasound in children within 5 months of hospital discharge. Only one patient of the 28 studied did not show normalization. Concomitantly, this patient suffered from cystic fibrosis, which could have a significant impact on the lung ultrasound result (13). Bottino et al. suggested in a preliminary observation, that children with an asymptomatic or mild infection do not develop pulmonary sequelae in the medium-term period. They did not detect any pulmonary complication or abnormality in their cohort of 16 enrolled patients (14). We were able to confirm this result in our study.

Clofent et al. were able to show in their study of 352 adult long COVID patients with a long-term follow-up of 2–5 months that lung ultrasound could be implemented as a first-line diagnostic procedure in the treatment course (6). It was shown that the outcome of lung ultrasound in adults could produce good discrimination between patients with persistent abnormalities compared with high-resolution computed tomography (HRCT). However, in this population, Clofent et al. found a high rate of interstitial lung disease after acute COVID infection, probably based on the patients already having much more severe symptoms in the initial COVID infection than in our group, which had shown a mild acute COVID course. Hereby it remains unclear whether these results are transferable to children, who typically show no or only mild symptoms in their acute infection phase.

While there is a body of evidence of lasting lung injury following acute COVID infection in adults (15), the data for children is currently insufficient. In concordance with our results there seems to be no morphological changes of the lungs in children and adolescent patients at least in patients with only minor symptoms in the initial COVID.

However, it should be noted that lung ultrasound is not able to image central lung sections due to technical conditions (16). It is only possible to examine peripheral sections or show consolidations that reach the pleura, assess the pleura, and form a judgment based on artefacts (16). However, because acute COVID infection of the lung manifests mainly in the peripheral portions of the lung (17), we would also expect that the changes in post-acute or long COVID cases would be more likely to be found in the periphery, as is the case in adult patients with long COVID (4).

In comparison to other LUS studies in children and adolescents we described a delicate scheme of examination. But, in contrast with previous studies we applied a strict randomization and blinding regarding any personal information or picture content in assessment of the examinations. To our knowledge, in no other work before the evaluation of LUS were performed so strongly in a blinded manner. Despite this assessment, we were able to achieve substantial inter-reader reliability with a Coheńs kappa of 0.67. This shows that LUS in children is a very reliable diagnostic method with a good scoring system even for examiners with few experiences.

In our cohort, there was no significant correlation between clinical parameters (number of symptoms, pathological lung function or single parameters of the lung function, heartrate) and LUS results or single LUS features (B-lines, consolidations, or pleural irregularity). This could be due to the absence of pathological findings in our cohort. Because Denina et al., unlike us, additionally performed a longitudinal follow-up of their cohort of acute to post-acute COVID cases in their study, they were able to demonstrate a significant correlation between their ultrasound findings and clinical parameters (13).

This study has some limitations. First, spirometry data of the healthy control group was not collected, limiting the comparability to the long COVID group. Second, the number of study participants is not very large, which limits the statistic power. In this study, no additional imaging such as x-ray or CT was performed, so no comparison was possible. For radiation safety reasons, a CT scan, which is the imaging gold standard in the diagnosis of interstitial lung disease (18), was not used. Recent work on hyperpolarized xenon 129 MRI shows very interesting results in non-hospitalized post-COVID-19 condition participants with normal chest CT examinations. Grist et al. were able to show in a prospective study with 11 adult participants that there are significant differences in lung function in comparison of healthy control and post-hospitalized COVID and non-hospitalized long COVID participants (19). Kooner et al. assessed 129Xe-MRI ventilatory defects, lung function, quality of life and exercise capacity in a total of 76 adult patients who had COVID-19 with persistent symptoms. They could demonstrate that the 129Xe MRI ventilatory defect percentage was significantly worse in ever-hospitalized compared with never-hospitalized participants and was related to the 6-minute walk test and SpO_2_ on exertion, but not to quality-of-life scores (20). These results are still fresh and further-reaching conclusions are therefore premature. Ultra-short echo-time MRI may present itself as a radiation-free alternative for lung assessment in children (21).

Our results might enable researchers to better assess the risk of long-term outcomes associated with COVID-19 and physicians to better advise and treat their patients (22).

## Conclusion

We could not detect any pathological findings in children and adolescents with long COVID on lung ultrasound. Based on our Study there seems to be no use for LUS in follow-up of Long-COVID Patients without initial pneumonia or pathological findings in an existing baseline Examination. Better standard Examination protocols need to be established for this patient group. Nevertheless, lung ultrasound is an important diagnostic tool for the lung, especially in children and adolescents, because of its radiation-free nature, rapid availability, and increasing establishment in practice and with increasing experience of the examiners.

## Data Availability

The original contributions presented in the study are included in the article/Supplementary Material, further inquiries can be directed to the corresponding author.

## References

[1] Nittas V Gao M West EA Ballouz T Menges D Wulf Hanson S Long COVID through a public health lens: an umbrella review. Public Health Rev. (2022) 43:1604501. 10.3389/phrs.2022.1604501 35359614 PMC8963488

[2] Zimmermann P Pittet LF Curtis N. How common is long COVID in children and adolescents? Pediatr Infect Dis J. (2021) 40(12):e482–e7. 10.1097/INF.0000000000003328 34870392 PMC8575095

[3] Wang Y Dong C Hu Y Li C Ren Q Zhang X Temporal changes of CT findings in 90 patients with COVID-19 pneumonia: a longitudinal study. Radiology. (2020) 296(2):E55–64. 10.1148/radiol.2020200843 32191587 PMC7233482

[4] Solomon JJ Heyman B Ko JP Condos R Lynch DA. CT Of post-acute lung complications of COVID-19. Radiology. (2021) 301(2):E383–E95. 10.1148/radiol.2021211396 34374591 PMC8369881

[5] Goodman TR Mustafa A Rowe E. Pediatric CT radiation exposure: where we were, and where we are now. Pediatr Radiol. (2019) 49(4):469–78. 10.1007/s00247-018-4281-y 30923878

[6] Clofent D Polverino E Felipe A Granados G Arjona-Peris M Andreu J Lung ultrasound as a first-line test in the evaluation of post-COVID-19 pulmonary sequelae. Front Med. (2021) 8:815732. 10.3389/fmed.2021.815732 PMC8794580 35096906

[7] Oks M Cleven KL Cardenas-Garcia J Schaub JA Koenig S Cohen RI The effect of point-of-care ultrasonography on imaging studies in the medical ICU: a comparative study. Chest. (2014) 146(6):1574–7. 10.1378/chest.14-0728 25144593

[8] Shah W Hillman T Playford ED Hishmeh L. Managing the long term effects of COVID-19: summary of NICE, SIGN, and RCGP rapid guideline. Br Med J. (2021) 372:n136. 10.1136/bmj.n136 33483331

[9] Volpicelli G Elbarbary M Blaivas M Lichtenstein DA Mathis G Kirkpatrick AW International evidence-based recommendations for point-of-care lung ultrasound. Intensive Care Med. (2012) 38(4):577–91. 10.1007/s00134-012-2513-4 22392031

[10] Volpicelli G. Lung ultrasound B-lines in interstitial lung disease: moving from diagnosis to prognostic stratification. Chest. (2020) 158(4):1323–4. 10.1016/j.chest.2020.05.528 33036082 PMC7533744

[11] de Jongh F. Spirometers. Breathe. (2008) 4(3):251–4.

[12] Schmidt KG Beyer C Hausler HJ Hofbeck M Redel D Vogel M Reports by the German society of pediatric cardiology. Quality standards for echocardiography in children and adolescents. Recommendations by the German society of pediatric cardiology for echocardiography studies in childhood and adolescence. Z Kardiol. (1999) 88(9):699–707. 10.1007/s003920050350 10525935

[13] Denina M Pruccoli G Scolfaro C Mignone F Zoppo M Giraudo I Sequelae of COVID-19 in hospitalized children: a 4-months follow-up. Pediatr Infect Dis J. (2020) 39(12):e458–e9. 10.1097/INF.0000000000002937 33003103

[14] Bottino I Patria MF Milani GP Agostoni C Marchisio P Lelii M Can asymptomatic or non-severe SARS-CoV-2 infection cause medium-term pulmonary sequelae in children? Front Pediatr. (2021) 9:621019. 10.3389/fped.2021.621019 34084763 PMC8168403

[15] Huang C Huang L Wang Y Li X Ren L Gu X 6-Month consequences of COVID-19 in patients discharged from hospital: a cohort study. Lancet. (2021) 397(10270):220–32. 10.1016/S0140-6736(20)32656-8 33428867 PMC7833295

[16] Pereda MA Chavez MA Hooper-Miele CC Gilman RH Steinhoff MC Ellington LE Lung ultrasound for the diagnosis of pneumonia in children: a meta-analysis. Pediatrics. (2015) 135(4):714–22. 10.1542/peds.2014-2833 25780071 PMC9923609

[17] Wang JG Mo YF Su YH Wang LC Liu GB Li M Computed tomography features of COVID-19 in children: a systematic review and meta-analysis. Medicine. (2021) 100(38):e22571. 10.1097/MD.0000000000022571 34559092 PMC8462638

[18] Sverzellati N. Highlights of HRCT imaging in IPF. Respir Res. (2013) 14(Suppl 1):S3. 10.1186/1465-9921-14-S1-S3 23734841 PMC3643237

[19] Grist JT Collier GJ Walters H Kim M Chen M Abu Eid G Lung abnormalities depicted with hyperpolarized xenon MRI in patients with long COVID. Radiology. (2022) 305(3):709–17. 10.1148/radiol.220069 PMC9134268 35608443

[20] Kooner HK McIntosh MJ Matheson AM Venegas C Radadia N Ho T (129)Xe MRI ventilation defects in ever-hospitalised and never-hospitalised people with post-acute COVID-19 syndrome. BMJ Open Respir Res. (2022) 9(1):e001235. 10.1136/bmjresp-2022-001235 35584850 PMC9119175

[21] Renz DM Herrmann KH Kraemer M Boettcher J Waginger M Krueger PC Ultrashort echo time MRI of the lung in children and adolescents: comparison with non-enhanced computed tomography and standard post-contrast T1w MRI sequences. Eur Radiol. (2022) 32(3):1833–42. 10.1007/s00330-021-08236-7 34668994 PMC8831263

[22] Nasserie T Hittle M Goodman SN. Assessment of the frequency and variety of persistent symptoms among patients with COVID-19: a systematic review. JAMA Netw Open. (2021) 4(5):e2111417. 10.1001/jamanetworkopen.2021.11417 34037731 PMC8155823

